# *Pneumocystis jirovecii* pneumonia in a X-linked chronic granulomatous disease female carrier

**DOI:** 10.1016/j.idcr.2021.e01323

**Published:** 2021-10-27

**Authors:** Vasiliki Kalotychou, Demetrios Mermigkis, Maria G. Kanariou, Marianna Tzanoudaki, Vasiliki Epameinondas Georgakopoulou, Irene Kourbeti, George L. Daikos

**Affiliations:** a First Department of Medicine, National and Kapodistrian University of Athens, Laikon, General Hospital, Greece; b First Pulmonary Department, Sismanogleio General Hospital, Athens, Greece; c Department of Immunology-Histocompatibility, Specialized Center & Referral Center for Primary Immunodeficiencies-Paediatric Immunology, "Aghia Sophia" Children's Hospital, Athens, Greece

**Keywords:** Chronic Granulomatous Disease, X-linked carrier, X-chromosome inactivation, Neutrophils’ oxidative activity, *Pneumocystis jirovecii*

## Abstract

The X-chromosome linked (XL) female carriers of chronic granulomatous disease (CGD) are considered to have no risk for infection. Herein we present a female CGD XL-carrier who developed *Pneumocystis jirovecii* pneumonia and *Serratia marcescens* infection associated with age-related skewing of X-chromosome inactivation.

## Highlights

• *Pneumocystis jirovecii* pneumonia in a female Chronic Granulomatous Disease X-chromosome-linked carrier.
• Skewing of X-chromosome inactivation.
• Decline in neutrophils’ oxidative activity with age.

## Introduction

Chronic Granulomatous Disease (CGD) is a rare inherited disorder in which a defect in one of the nicotinamide adenine dinucleotide phosphate (NADPH) subunits results in failure of neutrophils to generate reactive oxygen intermediates [1]. NADPH is a complex enzyme consisted of five subunits, gp91^*phox*^, p22^*phox*^, p47^*phox*^, p67^*phox*^, p40^*phox*^ encoded by the underlying genes *CYBB, CYBA, NCF1, NCF2, NCF4* respectively. The most common form of CGD (70%) is the X-linked (XL) form caused by genetic defects in the *CYBB* gene and inherited by XL recessive mode [1]. Female carriers of XL- CGD possess one copy of the X-chromosome with the mutated *CYBB* gene and one copy of paternal X-chromosome with the wild-type *CYBB* gene. Due to the process of random inactivation of one X-chromosome (lyonization), only one X-chromosome is active in each cell, making female X-chromosome gene dosage equivalent to that of males [2]. Thus, female XL-carriers have dual neutrophil populations: neutrophils with inactivated the *CYBB* mutated X-chromosome that are able to generate normal respiratory burst and neutrophils with inactivated the normal X-chromosome which are unable to generate respiratory burst. The balance between normal and defective neutrophils may get disrupted with aging due to acquired skewing of lyonization and female carriers with predominance of defective neutrophils may display clinical manifestations similar to those observed in CGD [3]. Herein we present a female CGD XL-carrier who developed *Pneumocystis jirovecii* pneumonia (PJP) and *Serratia marcescens* infection.

## Case presentation

A 34­year­old female, mother of a boy with XL-CGD, was admitted to the hospital with fever (38.5 °C), non-productive cough and difficulty in breathing. Computerized tomography of the chest showed infiltrates in both lower lobes. Antimicrobial treatment was initiated with ceftriaxone and azithromycin. On the 4th hospital day, the patient’s clinical condition was not improving and she underwent bronchoscopy with bronchoalveolar lavage. Immunofluorescence staining of bronchoalveolar lavage was positive for *Pneumocystis jirovecii*. Results of serologic tests for HIV-1 and HIV-2 were negative. Leukocytes, lymphocytes subpopulations, levels of immunoglobulins, including IgG subclasses, and complement components C3, C4, CH50 were within normal limits. Trimethoprim­sulfamethoxazole (TMP-SMX) was initiated and the patient had complete resolution of clinical symptoms and radiographic improvement. She was discharged home on secondary prophylaxis with TMP-SMX. One year later, six months following discontinuation of TMP-SMX, she presented with an abscess in the right thigh caused by *Serratia marcescens* which was treated successfully with drainage and administration of ciprofloxacin.

**Fig. 1:**
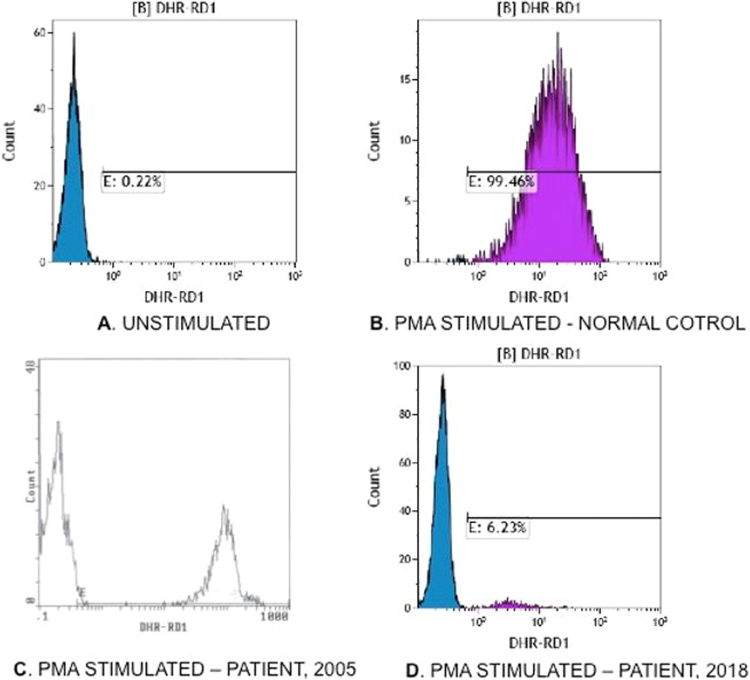
**Flow cytometry plots from the dihydrorhodamine assay**. Unstimulated (**A**) and PMA stimulated (**B**) neutrophils from a normal control. Patient’s neutrophils exhibited the typical CGD XL- carrier bimodal distribution upon PMA stimulation; in 2005, 34% of neutrophils generated normal oxidative activity **(C)**, in 2018, 6% of neutrophils had normal oxidative activity **(D)**.

In order to answer the question why this patient developed PJP and *Serratia marcescens* infection, we evaluated the oxidative activity of her neutrophils during the acute pulmonary infection and after convalescence. The flow cytometric dihydrorhodamine (DHR) assay revealed that only 6% of her neutrophils were able to mount normal oxidative burst, while at the age of 21 years, when her son was diagnosed with CGD, the DHR assay had revealed normal oxidative activity in 34% of her neutrophils (Fig. 1). Sanger sequencing confirmed the presence of C > T (C469T), which had been detected in her son. *CYBB* gene expression experiments revealed 80% decrease of the corresponding mRNA in the patient’s lymphomonocytes and granulocytes relative to ten age-matched healthy females. In addition, extreme skewed lyonization was demonstrated by the HUMARA (human androgen receptor gene) assay [4]. In particular, in DNA extracted from peripheral blood lymphomonocytes and granulocytes it was estimated that 86% of the paternal X-chromosome had been inactivated by methylation.

## Discussion

This case demonstrates that XL-CGD female carriers may acquire extreme skewing of X-chromosome inactivation with aging resulting in dramatic decrease of normally functioning neutrophils and become susceptible to bacterial and fungal infections. Indeed, in our patient, normal oxidative burst was produced by 34% of neutrophils at the age of 21 years, when her son was diagnosed with CGD, and this declined to 6%, 13 years later, at the age of 34 (Fig. 1). The reduced oxidative activity of patient’s neutrophils corresponded well to the skewing lyonization ratio of active to inactive X-chromosomes and to reduced expression of the *CYBB* gene as documented by the HUMARA assay and the expression experiments respectively. Although prior X-chromosome inactivation pattern was not available for comparison, apparently, progressive skewing of lyonization occurred with age, which resulted in loss of normally functioning neutrophils and as consequence in PJP and *Serratia marcescens* infections. Acquired skewing of lyonization may occur in females with aging and it may be a consequence of stem cell depletion, epigenetic factors, or other causes [5], [6].

While virtually all clinical manifestations of CGD have been described in XL-carriers, the majority of case reports and case series are referring to skin manifestations and autoimmune phenomena [3]. Despite the reduced oxidative activity of neutrophils in several XL-CGD carriers, recurrent infections have not systematically reported as a significant problem. Extreme skewing of lyonization, however, and dramatic decrease in the percentage of normally functioning neutrophils might increase the risk of infection as has been indicated by others and further supported by the present case [6], [7], [8]. In an attempt to assess which carriers are at risk to develop infections, Marciano et al. undertook a comprehensive analysis of data from a large cohort of XL-carriers and their findings suggested that the risk of infection increases when the percentage of neutrophils with normal oxidative activity drops to< 20% [8].

The majority of infections associated with CGD are caused by a limited number of organisms including *Staphylococcus aureus*, *Burkolderia cepacia*, *Serratia marcescens*, *Nocardia*, and *Aspergillus* spp [9], [10]. Although the number of reported infections in XL-carriers is quite small, it appears that the pathogens affecting the carriers are similar to those observed in CGD patients [3]. *Pneumocystis jirovecii*, however, one of the infecting organisms in our patient, very seldom causes infections in CGD patients and only a handful number of cases have been reported in the literature [11], [12], [13]. It is possible the lifelong antibacterial prophylaxis with TMP-SMX, which is administered invariably in patients with CGD, reduces the incidence of *Pneumocystis jirovecii* infections in this population.

In conclusion, the present report emphasizes the need for periodic clinical evaluation of all CGD female carriers and the importance to monitor their neutrophils’ oxidative capacity as it may decline over time and increase the risk of infection.

## Ethical approval

Informed consent was obtained from the patient for this report.

## Funding source

This work was supported by the special account for research grants of National and Kapodistrian University of Athens10.13039/501100005187.

## CReditT authorship contribution statement

V kalotychou: Molecular experiments and writing first draft; D Mermigkis: Data collection and writing, M G Kanariou and M Tzanoudaki: Oxidative activity experiments and manuscript editing, V Georgakopoulou and I Kourbeti: Data collection and review of the literature, G L Daikos: Study design and editing.

## Conflict of interest

The authors have no conflict to declare.
